# Communication, Collaboration and Care Coordination: The Three-Point Guide to Cancer Care Provision for Aboriginal and Torres Strait Islander Australians

**DOI:** 10.5334/ijic.5456

**Published:** 2020-06-08

**Authors:** Audra de Witt, Veronica Matthews, Ross Bailie, Gail Garvey, Patricia C. Valery, Jon Adams, Jennifer H. Martin, Frances C. Cunningham

**Affiliations:** 1 Menzies School of Health Research, Brisbane Queensland, Charles Darwin University, Darwin Northern Territory, AU; 2 QIMR Berghofer Medical Research Institute, Brisbane Queensland, AU; 3 University Centre for Rural Health, University of Sydney, New South Wales, AU; 4 Faculty of Health, University of Technology Sydney, Sydney New South Wales, AU; 5 School of Medicine and Public Health, University of Newcastle, Callaghan New South Wales, AU; 6 Southside Clinical School, University of Queensland, Brisbane Queensland, AU

**Keywords:** Indigenous people, cancer care coordination, integrated care, collaboration, communication, primary health care

## Abstract

**Aim:**

To explore health professionals’ perspectives on communication, continuity and between-service coordination for improving cancer care for Indigenous people in Queensland.

**Methods:**

Semi-structured interviews were conducted in a purposive sample of primary health care (PHC) services in Queensland with Indigenous and non-Indigenous health professionals who had experience caring for Indigenous cancer patients in the PHC and hospital setting. The World Health Organisation integrated people-centred health services framework was used to analyse the interview data.

**Results:**

Seventeen health staff from six Aboriginal Community Controlled Services and nine health professionals from one tertiary hospital participated in this study. PHC sites were in urban, regional and rural settings and the hospital was in a major city. Analysis of the data suggests that timely communication and information exchange, collaborative approaches, streamlined processes, flexible care delivery, and patient-centred care and support were crucial in improving the continuity and coordination of care between the PHC service and the treating hospital.

**Conclusion:**

Communication, collaboration and care coordination are integral in the provision of quality cancer care for Indigenous Australians. It is recommended that health policy and funding be designed to incorporate these aspects across services and settings as a strategy to improve cancer outcomes for Indigenous people in Queensland.

## Introduction

The coordination of cancer care for Indigenous Australians has been recognised as critical in addressing Indigenous patients’ needs, for example in relation to navigating the health system, providing essential information and communication and ensuring cultural safety [1]. However, challenges to the provision of coordinated care to meet Indigenous cancer patients needs include: lack of communication between services; delays in receiving timely hospital information; language and cultural barriers; distance to treatment; as well as providing cultural support and having hospital facilities that accommodate extended families [1,2,3,4]. These challenges often stem from health system design, structures and limitations [1].

Quality cancer care for Indigenous Australians as defined by the National Aboriginal and Torres Strait Islander Cancer Framework is evidence-based, person-centred (incorporating the family and cultural roles) [5,6], timely, equitable and delivered as close to home as safely as possible [5,7,8]. It is also multidisciplinary, integrated across the health sector (primary, secondary and tertiary), embedded within the community [5,9] and also involves strong involvement and leadership from the Indigenous community [5,10].

Pathways for accessing cancer care for Indigenous Australians can be more complex than for other Australians, with additional challenges for them relating to culture, language, and lack of familiarity with navigating services and institutions in the wider health care system [11]. Besides coming to terms with a cancer diagnosis, accessing diagnostic and cancer treatment often involves travel and being away from home for extended periods of time [11]. Cancer care also often involves accessing multiple healthcare providers (e.g. radiation oncologists, surgeons, pathology services) and across a range of settings [11]. Further, socio-economic impacts of accessing cancer care may also need to be considered [12].

Indigenous Australians have higher age-standardised incidence and mortality rates for all cancers combined, are less likely to be hospitalised following a cancer diagnosis and are less likely to survive five years after diagnosis (48% vs. 59%) compared to other Australians [13,14]. These disparities are due to a range of complex and inter-related factors including but not limited to Indigenous people being diagnosed at a younger age and with more advanced cancer, higher number of co-morbidities [15], a reduced uptake of health services, and barriers to accessing health services [1,16]. Experiences related to an enduring legacy of colonization, forced removal of children, racism, discrimination and loss of identity are examples of issues that continue to impact on the health and well-being of Indigenous Australians [17].

Health systems built on the principles of primary care, (the first point of contact and the provision of comprehensive and coordinated care) were found to achieve greater equity and better health outcomes compared to systems that focused on providing specialist care [18,19]. In Australia, over the past 45 years, Aboriginal Community Controlled Health Services (ACCHSs) established and governed by local Indigenous communities, provide culturally competent comprehensive primary health care (PHC) [20] to meet the local Indigenous community needs [21]. ACCHSs have successfully reduced barriers including unintentional racism, and improved access to care for Indigenous Australians [22]. One recent qualitative study reported the success of a cancer care team based at an ACCHS that helped streamline services, improve cancer service accessibility and provide culturally safe care [23].

### WHO Integrated people-centred health services framework

The World Health Organisation (WHO) developed a framework on integrated people-centred health services (IPCHS) recommending a fundamental shift of health systems from being disease focused to people-focused [24,25]. This approach promotes equity in access to healthcare, is responsive to people’s needs, and strengthens the capacity of health systems [25]. The framework identifies the primary drivers of continuity and coordination of care [26]. Continuity of care promotes an environment to develop ongoing relationships that support seamless interactions between service providers within and across sectors that enable coordination of care [26].

Care coordination can be viewed as a broader strategy to improve care by bringing together providers and professionals to meet service users health needs [26] and delivering integrated, person-centred care across settings [26,27]. Together, continuity and coordination of care are vital to deliver quality care and are likely to be important in improving cancer outcomes for Indigenous patients [28]. Although there is growing knowledge on this topic from the patients’ perspective, it is also important to understand the health professionals’ perspectives. As health professionals are more likely to be familiar with the inner workings and intricacies of the health system, they are likely to be well positioned to identify gaps in care across the cancer trajectory and areas for improvements.

The aim of this paper is to explore PHC and tertiary hospital professionals’ perspectives on issues related to communication, continuity and coordination of care between PHC and hospital services and to recommend strategies for improving the delivery of cancer care for Indigenous people. We draw upon the IPCHS framework to analyse the interview data, applying the framework’s primary drivers of continuity and coordination of care. This paper presents findings from a study which forms part of a larger cross-sectional study investigating the patterns of care of Indigenous cancer patients in Queensland [29,30,31,32].

## Methods

This study used a concurrent qualitative design, based on health professional interviews employing semi-structured guides. Participants were purposively sampled from several ACCHSs in urban, regional and rural geographical locations, and from a large urban cancer treating hospital in Queensland [33]. Interviews were conducted with Indigenous and non-Indigenous health professionals experienced in providing care for Indigenous cancer patients. This qualitative methodology enabled the in-depth, rich exploration of direct feedback from health professionals on the provision of cancer care to Indigenous patients based on their experiences [34]. The qualitative findings from this study will inform the body of work from the larger cross-sectional study.

### Data Collection/Procedure

Staff from participating health services were invited to participate in interviews. The researchers sent an email invitation to the health service manager at participating sites outlining the study criteria. Following this, the service manager invited their health staff that met the study criteria to participate in interviews. Not all invited health staff chose to participate in the study. Participant interviews took place from May 2015 to August 2016 at locations and times suitable for participants. A semi-structured interview guide was developed, piloted and refined. Open-ended questions explored health professionals’ perspectives about follow-up cancer care for Indigenous patients attending the PHC service, hospital and the interface between the PHC and tertiary setting. This interview format allowed for further investigation of relevant issues as they emerged during interviews [35]. Interviews explored participants’ perspectives of the continuity, coordination of care and extent of communication between treating services, and the timeliness of information received from treating services. Participants were then asked to recommend strategies to improve the delivery of continuous and coordinated cancer care. All interviews were conducted face-to-face except for one telephone interview and were audio-recorded with participants’ consent. Data collection ceased when data saturation was achieved [36] and as agreed upon by AdW and FCC. Data saturation was reached at interview number 17 for PHC participants (17 interviews conducted) and interview number nine for hospital participants (Nine interviews conducted). All participants were provided the opportunity to review their interview transcript. The interviewer (AdW) was unknown to all participants prior to the interviews.

### Data analysis

Interview recordings were de-identified, professionally transcribed and imported into NVivo (version 10) for data management and to assist with analysis. Thematic and inductive analysis was conducted involving an iterative process of data coding, reviewing an initial list of codes (AdW, FCC, AL), organising codes within themes, revising themes against codes for consistency and completeness until key themes were identified [37]. Once key themes were finalised, interview data were then categorised and analysed utilising the WHO IPCHS framework [26]. A three-step process for coding semi-structured interviews was utilised to ensure a high level of intercoder reliability [38]. The initial stage involved two researchers independently analysing a sample of manually coded transcripts (AdW and AL). These constructed codes were then compared for any inconsistencies and for intercoder reliability [39]. The second stage involved discussing coding differences and disagreements. There were no coding disagreements, and a high level of intercoder agreement was established (AdW, AL, FCC). The final stage involved utilising agreed codes on the full set of transcripts. A high level of inter-coder reliability, along with inclusion of participant quotes, contributed to research credibility and analytic rigour [38,39]. The consolidated criteria for reporting qualitative research (COREQ), a checklist to report on important aspects of a qualitative study, was used to guide the reporting of our findings [40].

### Ethics approval

This research was approved by the Human Research Ethics Committees of the Darling Downs Hospital and Health Service (HREC/14/QTDD/32), Menzies School of Health Research (HREC/2014-2222), QIMR Berghofer Medical Research Institute (HREC/P2127) and by the relevant Aboriginal community boards of participating services.

## Results

Six ACCHSs out of the seventeen services invited agreed to participate in the health professional interviews. Seventeen health professionals from the ACCHSs, and nine health professionals from a tertiary hospital were recruited in Queensland. (See Table 1 for participant characteristics). Interviews were on a voluntary basis. Not all health professionals at participating sites were interviewed. The average interview duration time was 56 minutes across both sites.

**Table 1:** Participant characteristics.

	Total number of participants in settings		Self-identified Indigenous participants

	PHC	Tertiary	

***Sex***			
Male	6	3	
Female	11	6	
***Profession***			
Receptionist/admin officer	1		1
Aboriginal Health Workers/Professionals	2		2
Aboriginal Liaison Officer		2	2
Allied Health Coordinator	1		1
Maternal and Child Health Manager	1		1
Enrolled Nurse	2		1
Registered Nurse	4	1	3*
Nutritionist		1	–
Social Worker		1	–
General Practitioner	6		–
Medical Oncologist		1	–
Radiation Oncologist		2	–
Hematologist		1	–
***Total number of health facilities and geographical location***			
Urban area	1	1	
Inner regional	1		
Outer regional	3		
Remote area	1		

Key: * denotes PHC setting.

Based on the analysis of the data, the key themes (major findings) relating to the continuity and coordination of care in this study included: communication and information exchange; collaborative approaches to cancer care; streamlined processes to enable the sharing of patient information; flexible care delivery and patient-centred care and support. Enablers, barriers and strategies of key themes were categorised using the WHO IPCHS framework of identified primary drivers of continuity and coordination of care (Table 2). The full list of participant recommended strategies to improve continuity and coordination of care and communication between treating services is provided in Tables 2 and 3 respectively. Recommended strategies are predominantly categorised under one primary driver heading only, even though strategies may be relevant in addressing barriers listed in other categories. For example, the PHC recommendation that the hospital provides notification to them of patients’ hospital admission and treatment updates is relevant to both informational and cross-boundary team continuity but, is listed under cross-boundary team continuity category only (Table 2).

**Table 2:** Enablers, barriers and strategies for improvement in the continuity and coordination of care within and between the PHC and cancer treating tertiary hospital as identified by study participants, displayed in IPCHS categories.

WHO IPCHS identified primary drivers of continuity and coordination of care	Enablers	Barriers	Strategies for improving continuity and coordination of care

**Interpersonal continuity** (refers to patients’ experiencing continuity in their trusted therapeutic relationships, care provided based on identified personal and cultural needs provided by a central provider) [26,41].	Continued relationships and trust Patient follow-up and provision of holistic care General practitioner (GP) as central point	Staff workload resulting in minimum time for patients (hospital setting) Limited staff knowledge to provide adequate care	**Education/Upskilling of staff.** PHC staff – education on diagnosed cancers, treatment and management. Hospital staff – education on health and hospital associated needs of Indigenous patients. **Patient centred care.** Aboriginal Liaison Officer (ALO) providing cultural support and care prior to patient presentation to hospital based on patient-identified needs, use of translators as required **Collaborative practices** and proactive attitudes/practices, peer support amongst care providers within and between sectors
**Longitudinal continuity** (refers to patients seeing the same professional over multiple episodes of care, ensuring strategies are in place for care to be connected, and availability of a patient support network) [26,41].	Proactive approach to patients care Patient navigator Patient support to access services	Systems and processes resulting in barriers to care e.g. organisation protocols resulting in a lot of paperwork and time delays)	**Practical support** for patients to access care (for example, transport, accommodation, care coordinators/navigators **Collaborative practices** across settings to plan patient’s post-discharge care and follow-up prior to hospital discharge **Sharing of electronic patient records** across hospital and PHC sites **Seeing the same care navigator/coordinator through the cancer journey** across settings to deliver care continuity **Simpler protocols around release of patient information documents** for treating PHC services to obtain copies of patient hospital records to be able to provide care continuity and quality follow-up care at the PHC end
**Flexible continuity** (refers to the ability to adjust care and treatment plans in response to patients changing individual needs across time [26].	Flexible care delivery (e.g. telehealth) ‘Drop-in’ clinics Extension of clinic hours (PHC) Longer GP/specialist patient consultations Visiting specialists/allied health professionals at PHC setting	Rigid hospital appointment schedules/times Transport and parking costs (hospital setting) Short hospital consultation times with specialists Lengthy waiting times before seeing specialist (hospital setting)	**Care flexibility (especially at the hospital)** and consideration of individual patient needs when scheduling appointments (for example, travel distance), in care delivery (such as telehealth options), availability of outside business hours clinics to access allied health staff, practical considerations (for example, parking costs, public transport). **More consideration for appointment flexibility for patients living outside urban areas** **More time allocated for specialists’ consultations** **Active follow-up of patients** who are unable to attend scheduled appointments **Patient support** (help with self-management strategies and to prioritise health as needed) **Timely and improved communication pathways between services** **Keeping patients informed** whilst waiting to be seen in hospital
**Informational continuity** (refers to the provision of timely and comprehensive information in relation to patient care needs) [26].	Timely communication and information exchange Use of technology	Limited communication & coordination/teamwork within hospital Delayed communication and information exchange on patient treatment/condition Ineffective administrative/system processes (such as some PHC having difficulty accessing paperwork)	**Sharing of patient electronic records** across settings in real time **Timely, frequent and ongoing communication** and more use of telephone calls, case conferences between services, specialists providing updates to GP after every hospital specialist appointment (not only for changes and/or to discuss concerns) **Name and contact details of a contactable medical staff member** as point of contact on all discharge summaries **Patient information** – ensuring patients are aware they can access both hospital and PHC care services after hospital discharge **Streamlined administrative processes and paperwork** (for example, streamlined release of patient information consent forms)
**Cross-boundary team continuity** (refers to effective collaborations among professionals in all care settings) [26].	Collaborative partnerships, teamwork and good relationships within and external to the organisation Care management plans	Working in silos Lack of clarity between PHC and hospital staff on who’s providing the follow-up care Lack of streamlined services and system processes	**Working together, good relationships and follow-up care.** Hospital providing discharge summaries and/or paperwork to PHC in timely manner, shared patient management care plans, developing communication protocols between services, more collaboration across multidisciplinary teams in hospitals **Shared patient electronic records** across services in real time **Clarity around professional roles and responsibilities** (for example, role of ALO and social worker at the hospital) and clarity between hospital and PHC staff on post discharge follow-up care (who is doing what) **Communication/engagement with PHC** such as copying GP in relevant communications with specialists, referrals, keeping PHC in the loop **Prompt follow-up of positive test results** and communication with GP and relevant professionals **Hospital admission notifications and treatment updates to PHC** while patient is hospitalised (with patient consent) **Streamlined administrative processes** **Designated site contact person** at the PHC and hospital is provided to services as a point of contact for any patient and care related enquires

**Table 3:** Study participants recommended strategies on improving communication between the treating hospital and PHC services.

Themes	Participants recommendations	Sample Hospital participant quotes	Sample PHC participant quotes

**Shared patient records/care plans, use of technology**	**Hospital and PHC:** Increase use of phone calls to discuss patient care in timely manner, more timely information exchange, sharing of electronic patient records and use of other technology (e.g. telehealth, teleconferences) to discuss patient care in a timely manner. **PHC:** Prompt follow-up of urgent concerns, clearly documented and shared cancer treatment and management plans.	“It’s about timeliness of the delivery of information and I do think that often there’s an unacceptable delay between the patient’s diagnosis and the GP getting the information, because the patients have very commonly been back to see their GP before the GP has received a letter from us.” (Hospital 6, Non-Indigenous specialist)	“So, maybe if it’s consented (to share patient’s hospital records) when they (patients’) first go in… then after that’s fine. So, if paperwork has come, then they can just send it straight through… if the consent is given for the doctor to share the paperwork while our patient is still in hospital, then that might be a good thing too… because then too once again… if a doctors have got a heads up about what’s happening and what needs to be done when the patient comes out, and then they can start to organise that before they come out.” (PHC 8, Indigenous EEN)
**Working collaboratively, clarity around follow-up care**	**Hospital and PHC:** Having a designated contact person at each site and associated contact phone number for health professionals to contact to discuss patients care. **PHC:** Clarity around post-hospital discharge follow-up care (e.g. who is doing what and when), development of agreed communication protocols and processes between services, regular face-to-face meetings to discuss patient care/needs and collaborative action to address/support patients prior to hospital discharge. **Hospital:** Hospital ALO’s to engage in more community engagement activities with PHC services and community, ensuring hospital has correct GP contact details for communication	“I know at one point there we were doing a lot of community engagement so we were able to actually leave the hospital and go and visit organisations and things, but we kind of cut back on that community engagement. I suppose if we returned to that level of community engagement again that would probably help.” (Hospital 8, Indigenous ALO)	(Regarding discharge summary paperwork) “there’s actually someone (named on discharge summary) that can be contacted … so, for instance I may not know who the registrar is, and within Queensland Health they will change during a year, so for there to be a named individual on the discharge summary that says, ‘if you’ve got any questions please phone blah blah blah, and here is my mobile number’, so that there’s a named point of contact.” (PHC 3, Non-Indigenous care coordinator RN)
**Notifications/updates around hospitalisations, accurate GP details**	**PHC:** Notification from hospital to inform treating PHC of patient admission, discharge and regular updates on patient condition during hospitalisation. **Hospital:** having updated treating GP information for the patient.	“Again, it will come down to as long as we’ve got the right GP and that the patient is going back there. I think if we see a patient and we get them through the process and treat them, we make sure that we explain to them as best we can what the recovery is going to be, we do a follow-up phone call which I do… and then send those letters to the GP. I think that’s pretty good communication and always know or trusting that a Doctor would call if they need to.” (Hospital 5, Non-Indigenous RN)	“It’s just… I spose ongoing communication. What I’d like to see is more information about patients who are in the hospital… so we know who is in the hospital. Whether they’re in there for… say they got sick at home and they’re now in having treatment at palliative care. I’d like to know about all our patients… all Aboriginal patients that may be in palliative care. I’d like to know as soon as… not wait two weeks down the track.” (PHC 9, Non-Indigenous social worker)
**More efficient administrative processes**	**PHC:** Hospital obtaining patients signed consent once (rather than multiple times) to release hospital treatment records to the treating PHC. **Hospital:** Reducing the turnaround time for hospital specialist letters to be dictated and sent to GPs, use of electronic systems	“We need to be emailing these letters and they need to be going electronically to GP’s, but at an administrative level.., I think that we need to improve those communication pathways, and it needs to be electronic.. but then, there’s still this delay in the dictation process.” (Hospital 6, Non-Indigenous specialist)	“Oh, it’s just I suppose it’s just the feedback. When they’ve seen the specialist you need to have that feedback from the specialist. It’s just this paperwork that you need to chase up all the time. Especially when they get into those big hospitals.” (PHC 13, Non- Indigenous EEN)

### Interpersonal continuity

Continued and trusting relationships with health providers, adequate patient follow-up care, and the general practitioner (GP) as the central point of care are examples of participant-identified enablers for continuity and coordination of care in this study.

One nurse participant shared:

“I think it’s really important that the partnership is formed between their treating doctor and the hospital. I think that’s very important. Because at least we know that our treating doctor will be that person that has continuity of care that is aware of all of these things.” (PHC 10, Indigenous Maternal and Child Health Manager)

Another PHC participant from a regional setting explained that cancer patients were often seen by different doctors each time they attended a public hospital and thus spoke of the importance of adequate follow-up care provided by the PHC service which provided care continuity for patients:

“We work with them (Indigenous patients) constantly. You don’t just let them walk out the door and say, ‘see you next time’. We always ring, and because it’s close here too… we’ll just go out and see them and check up on them.” (PHC 14, Indigenous Registered Nurse) [RN]

Participant-Identified barriers (across sites) included limited knowledge of some staff on qualitative study, was use cultural and clinical aspects of health care delivery for Indigenous people. This impacted on staff’s ability to provide quality cancer care and to meet the needs of Indigenous patients. One hospital participant informed:

“One thing that strikes me is that it may well be that the current generation of doctors are being taught at medical school about Indigenous issues, but the current crop of decisions makers are people who weren’t, either because that wasn’t part of the Australian Medical Education or they didn’t (receive) Australian Medical Education….. and now they’re in leadership positions and frankly we just haven’t been trained… and we want to be, we really do, but we haven’t been trained.” (Hospital 2, Non-Indigenous specialist)

### Longitudinal continuity

Participants identified the provision of patient support to access care, dedicated staff members, patient navigator style roles, and health professionals’ proactive attitudes as enablers to continuity and coordination of care in the Indigenous cancer context.

An Indigenous health worker (IHW) in a regional setting explained:

“I go with them, yes. I’ll take them up and I’ll book ‘em in and wait until they go into surgery and then I’ll go. If it’s just day surgery then I’ll come back later in the afternoon or just keep ringing them to see how they are and if they’re out of day surgery. So, yeah, that’s the support I give.” (PHC 17, Indigenous Health Worker) [IHW]

Hospital participants also acknowledged the importance of working with patients. One hospital professional shared:

“We work with him to facilitate him coming to hospital to get treatment… we’d ring them (the primary health care network) and say, ‘OK, such and such has got to be here on Friday, let’s round up the family and organise transport, we’ve gotta get accommodation ready for him and then have a few dry runs to try and get him down.’ If he’s coming on Friday then he probably wouldn’t come until Monday or Tuesday.” (Hospital 7, Indigenous Aboriginal Liaison Officer) [ALO]

Hospital staff reported that cancer care coordinators at this same hospital were similar to patient navigators providing examples of how to increase patient’s accessibility to services and facilitate care continuity at the hospital setting:

“Things like scheduling chemotherapy, booking in radiotherapy, making sure they got to their psychology appointment, ensuring that they got their pharmacy supplies… it’s all very practical stuff, and they’re only providing that care while they’re here (at hospital) on treatment. There’s no continuity of that care after they finish treatment.” (Hospital 3, Non-Indigenous allied health)

Several hospital staff spoke of the significant amounts of paperwork and time delays identifying these as barriers to care. One participant spoke of these in the context of travel applications and accommodation subsidies for patients travelling from outside urban areas for cancer treatment:

“Mmm… I’m mainly thinking about it in looking at streamlining in how fast it can happen to set-up, approve the (I’m talking about) patient travel subsidy scheme which is, you know, a subsidy that once they’re approved they get $66 per night towards accommodation, and if that can happen faster or be approved faster then there’s not so many hoops to jump through. The paperwork is… there’s gotta be… (an easier way).” (Hospital 8, Indigenous ALO)

### Flexible continuity

Flexibility in care delivery, drop in clinics, extension of clinic hours, longer GP and specialist consultation times were some participant-identified enablers to continuity and coordination of care in the Indigenous cancer context.

One hospital participant spoke of how flexible delivery of care was provided to cancer patients:

“So, a lot of these centres that have a big… you know, rural catchment area, they have these arrangements with smaller peripheral hospitals to try and provide chemotherapy using tele-health support.” (Hospital 6, Non-Indigenous specialist)

Another participant explained how the hospital avoids displacing patients from their homes and communities:

“…when we treat someone from a long way away, if we can get them followed up locally we try to. We only bring them down here if we really need to see them with our own eyes. If we can ask someone else to review them we’d much rather they’re at home.” (Hospital 4, Non-Indigenous specialist)

However, participants across sites acknowledged that patients sometimes needed to travel for cancer treatment and follow-up care, and this sometimes constituted a barrier to care, especially when patients needed to access multiple hospital services in one day as below:

“It’s enormously problematic for people from Indigenous communities to travel. There’s no right or wrong answer to that but it’s just a reality. A lot of Indigenous clients miss appointments and reviews. Video tele-conferencing facility and capacity is ideal in that circumstance where you know that the access to the regional centre may not occur.” (PHC 15, Non-Indigenous General Practitioner) [GP]

“The issues for a lot of these patients is that they might not be able to do their appointments on the same day. So, they might not be able to see us and the physio on the same day or us and the occupational therapist on the same day. So, I think there’s issues around accessing services at the same time as their appointment…. by the time a lot of the patients get seen, those additional services have left the hospital. They’re not available, because most of them leave by about 4.30 in the afternoon. If someone has got an appointment at 5.00 or 6.00 then it’s too late to say, ‘oh, we’ll get the dietician to come and see you,’ because they’ve gone for the day.” (Hospital 6, Non-Indigenous specialist)

### Informational continuity

In this study, timely communication, information exchange and use of technology were identified by participants as enablers for continuity and coordination of care.

One hospital specialist explained:

“So, when we’d seen the patient, we would send them (PHC) information about the nature of their diagnosis and the recommended treatment, a rationale for the treatment, what it would involve and the potential side effects. At the completion of treatment we would write again and let the GP know what treatment they’d been given and how the patient tolerated it and details of the patient’s follow-up appointment with us and what the plan is from here on in.” (Hospital 1, Non-Indigenous specialist)

In addition to the feedback above, several other specialists informed they would regularly send letters to PHC services for any changes to treatment or if there were issues of concern (for example, acute medical issues or social wellbeing concerns). For urgent matters one specialist informed:

“If I see a patient who I’m concerned about the delay in the time that they’d get the letter and I think there’s something that the GP needs to know more urgently, I would try and ring. Yeah. It’s sometimes hard because it’s often at the end of your working day that you’ll try and ring and they’re often not available. You know, they’ve often gone home, so sometimes it’s difficult to communicate with them in real time.” (Hospital 6, Non-Indigenous specialist)

One GP spoke of how communication with the hospital markedly improved after he lobbied and was successful in establishing a shared electronic medical record (EMR) system. He explained:

“…one of the key factors has been the sharing of electronic health records so both services have access to updated information for that client in that moment. That then enables them (even after hours) to phone the appropriate services in the regional centres (x, y) to access information and advice on treatment (depending on what the client’s needs are). We’ve never had any obstruction in terms of making those calls in or out of hours…[coordination of care between PHC and local hospital since sharing of electronic records has) 3,000% improved.” (PHC 15, Non-Indigenous GP)

PHC participants also spoke of challenges relating to obtaining information from the treating hospital and called for more streamlined processes for timely receipt of hospital information as delays in information could jeopardise patient care.

One participant noted:

“Sometimes there’s a delay in that (hospital sending discharage summaries), but that’s dependent on the hospital and the staff there. There are some parts of the hospital that are very efficient (sending discharge summaries electronically), while others mail it or fax it. It’s not a uniform thing as far as discharge summaries are concerned from hospitals.” (PHC 15, Non-Indigenous GP)

Several hospital-based participants explained they were aware that PHC services often experienced delays in receiving information from the treating hospital. One hospital participant suggested that the outsourcing of hospital dictation services to an overseas company, in combination with the changes to mail deliveries by Australia Post, had resulted in an increased delay in postal delivery time throughout Queensland. Several hospital participants informed they were in favour of sharing electronic patient records with the patients’ treating PHC services.

### Cross-boundary team continuity

In this study, good relationships, collaborative partnerships, and patient care plans were identified by participants as enablers for the continuity and coordination of care within the cross-boundary team continuity context.

Study participants spoke of the importance of teamwork between staff members across professional disciplines within organisations, as well as collaborative partnerships with external organisations. One regional GP advised that they had recently developed communication protocols with their local hospital to improve collaboration and care continuity:

“The main issue is the follow-up planning. I mean what works quite well which we’ve just recently started with the ED [executive director] is having regular meetings where somebody from our unit will go and have a discussion with them, and they would raise some of our patients which they have issues with… and we’d have a discussion about who is doing what (and so forth)…” (PHC 11, Non-Indigenous GP)

Several participants from both PHC and hospital sites also spoke of barriers such as hospitals working in isolation, and in combination with the lack of streamlined processes, this sometimes impacted on the continuity of patients’ care.

One participant shared:

“I guess we pretty much work in silos here so that each member of the allied health team is really quite familiar with their roles and responsibilities, but in terms of the specific details of other staff members we’re not quite sure how they work. Obviously we know how the consultants work because we see their letter in the chart, so we know that every GP has been sent a letter from the consultant.” (Hospital 3, Non-Indigenous allied health)

In regard to follow-up care, one GP shared:

“The follow-up… it is not only with oncology but other places that it regularly happens where the discharge summary will say, ‘follow-up organised with whoever’, and then we’re waiting for the appointment (and the patient is waiting), and then the appointment never happens… and then a month or two months down the way they still haven’t heard anything, and then you would phone that department and they would say, ‘oh, they don’t have a referral’. And we’re like, ‘it’s written in the discharge summary… they’ve organised the referral’. They didn’t ask us to refer the patient, and then in some way the internal referral system seems to not have happened. So, then I will say, ‘OK’, and then I do a referral.” (PHC 11, Non-Indigenous GP)

The issue of patients providing signed consent multiple times to release their hospital information to the treating PHC service, concerned several PHC staff as below:

“That’s my point… because we’ve already got …. the consent, and they’re seeing the same doctors… and it’s all this paperwork too that freaks them out. Like, you know… they’re forever thinking, ‘I’ve given my consent’. As far [as] they’re concerned they’ve given their consent… that’s it… end of story.” (PHC 8, Indigenous Endorsed Enrolled Nurse) [EEN]

Another PHC participant added:

“I would (for example) refer a patient to the hospital (not just for cancer care) and then the patient comes back and you don’t have the information… and you would phone the hospital and they would tell you that you need to get a signed patient consent form… and sometimes the patient isn’t here. So, then we have to go and find the patient and bring them in to sign a consent form…” (PHC 11, Non-Indigenous GP)

The GP informed this was inconvenient, involved additional time and resources, and most importantly, delayed providing timely patient follow-up care.

## Strategies for improving communication, continuity and coordination of care

Broadly, participant recommendations to improve continuity, coordination of care and communication between services centred around the provision of timely patient information from the hospital to the treating PHC service, clarity around hospital discharge follow-up care, sharing of patient records and care plans, services working more collaboratively, hospitals providing notifications and updates to PHC service during patient hospitalisation, and more efficient administrative processes. See Tables 2 and 3 for a full list of recommendations, and Table 3 for additional participant quotes.

### Communication, collaboration and care coordination

Recommendations from PHC and hospital participants centred around faster delivery of information and strategies to support this including the increased use of telephone calls, emails, shared EMRs and other technology (such as teleconferences). The need for continuing education and the upskilling of health staff was also evident (Table 2). Hospital staff also suggested a patient-centred care approach, the provision of cultural support and the use of interpreters for patients as required to address patient-identified needs. The extension of the role of the hospital ALO for community engagement activities, as was previously practised, was also recommended by an Indigenous hospital participant to promote collaborative links between services.

PHC participants highlighted the need for the hospital to provide clear instructions on the type of follow-up care required post hospital discharge and to explicitly state whether the hospital or PHC service was to complete the follow-up care. The development of appropriate communication protocols between the PHC and hospital service was suggested by a number of PHC participants to improve communication and collaboration. Providing the name and telephone contact details of a designated contact person at the treating services and having correct GP details on hospital records were also identifed as important to enhance communication, collaboration and care coordination.

### Streamlined processes, flexibility in care and patient support

Participants recommended improvements to the current hospital administrative processes to improve communication, continuity, and coordination of care between services. Hospital notification to the PHC service of a patient’s admission to hospital, and provision of regular patient updates to the PHC service while patients are hospitalised (with patient consent) were also recommended by several PHC participants. Both PHC and hospital participants spoke of the need for flexibility in delivering care to patients based on individual needs. Cancer care coordinators providing ongoing support across settings and throughout the cancer journey to provide care continuity and practical assistance such as with transport and parking costs was also suggested.

## Discussion

In this study, high levels of communication, timely information exchange, collaborative approaches to patient care, streamlined processes for efficient sharing of patient information, flexible methods to deliver care and patient-centred care and support were identified as the major facilitators in delivering continuous and coordinated care to Indigenous cancer patients. Key strategies that were identified, specifically relating to improving communication between services, included: shared patient records and care plans, and the use of technology; working collaboratively and having clarity around post discharge follow-up care; provision of information notifications around hospitalisations and accurate GP details, and more efficient administrative processes. Three central issues that study participants continued to reiterate related to: timely communication; care continuity and coordination and collaboration.

Participants in this study reported the lack of communication and timely information being received at the PHC service from the hospital as a core challenge in the provision of quality cancer care to patients. These findings are consistent with other studies which found that ‘fragmented communication’ referring to the breakdown in communication and information sharing including ineffective referral systems [42], was often found to be the central issue surrounding problems at the interface between primary and specialist services for cancer patients [4,42,43]. In a related study investigating Indigenous patients perspectives in follow-up cancer care, timely and relevant discharge information, continuity of care, good communication between service providers and strong therapeutic relationships were also identified as key issues in the provision of quality cancer care [30]. One Canadian study reported that system-levels challenges to cancer care related communication between family physicians and specialists included delays in medical transcription, difficulties accessing patient information and physicians not being copied on all reports [4]. The provision of timely information about treatment received at the hospital was critical for PHC services to provide quality follow-up care and help reduce the likelihood of patients being lost to follow-up, as services often had no prior knowledge of this, and it took time to obtain patients’ hospital records. PHC professionals reported that the need to re-consent patients each time their hospital information was requested to be released for PHC services to provide follow-up care also contributed to the delay in receiving timely information.

Participants highlighted the need for increased levels of continuity and coordination of care between services in order to deliver quality care to Indigenous patients across settings. The goal of continuity of care is to create seamless interactions across providers, within and between teams, organisations and settings, and in itself, continuity promotes care coordination [25]. Primary care and specialist care integration are both crucial to offset system fragmentation in the provision of comprehensive care and to improve continuous and coordinated care across settings and needs to be addressed at all levels including clinical, organisational and policy levels [44,45]. Fragmentation stems from the way health systems are designed to provide specialised, disease focused medical care (vertical integration) which is on the opposite integration spectrum of holistic comprehensive care [44,46]. An additional challenge in addressing system fragmentations is that continuity and coordination of care have been defined and measured in many ways which makes it challenging to find clear solutions to address these health system challenges [27,47]. However fragmented services are ineffective in meeting the needs of patients with chronic conditions [26,48] and, hence, this is the reason improved coordination of care has been on the agenda for health system reform in many countries [49].

Cancer care coordination is critical in facilitating and supporting a person during diagnosis, staging treatment planning, and delivery phases of care [4,11]. It can address fragmentation of care, poor communication between multiple providers, duplication of investigations and avoidable hospital readmissions [50]. When accessed, care coordination plays an important role in overcoming challenges patients may experience in accessing and engaging with the health system including cultural, social and practical barriers that often impede Indigenous patients’ access to the health care system [1]. It helps participants engage earlier with the health system [51], is especially helpful for patients with complex cancer diagnoses and psychosocial needs and those requiring input from multiple health providers, and has assisted the transition of patients back into the community upon hospital discharge [1,50]. In this study, several participants spoke of the importance of a patient navigator to help Indigenous patients with their cancer care. Patient navigators have previously been utilised, for example in two studies relating to American Indian tribes: by helping American Indian tribes navigate cancer therapy and the health care system [52] and in improving women’s access to breast cancer screening services (‘Native Sisters’ program) [53]. Given the ‘exceptionally high’ compliance rate with breast screening as reported in the study, it is likely that such a program could improve cancer mortality rates.

The need for streamlined administrative processes between the hospital and PHC services, collaborative approaches to improving patient outcomes such as shared EMRs and challenges around processes for the hospital release of patient information to the treating PHC service were highlighted in this study. Integrating health information is one approach to facilitate safe and effective health care delivery and at a lower cost [54]. The need for such streamlined approaches to address the lack of standardised electronic and communication platforms to share cancer patient records across service settings has also been recognised in other countries such as in Canada [55].

The Queensland government initiative to implement integrated EMR within state hospitals is currently in progress with identified benefits reported to include: availability of up-to-date patient information at the point of care; ability to share patient records electronically; safer and more reliable care; improved efficiency; more time spent with patients and less paper use [54]. Apart from one service, PHC services in this study did not have shared access to patients’ hospital EMR. As a patient’s cancer journey involves accessing multiple service providers across multiple settings, it is important that treating health professionals have point-of-care access to patient records to provide continuous, best-practice, coordinated care to increase the likelihood of improved patient outcomes [56,57,58,59,60].

Flexibility in the provision of care and practical support were also identified to be critical in increasing service accessibility for Indigenous cancer patients in this study. While a number of hospital participants recognised the need for flexible appointments in consideration of patients’ personal circumstances, it was not always possible to offer such flexibility due to the nature of how hospital systems and processes are currently organised (for example, many patients slotted into the same appointment time schedule to see the cancer specialist and the long wait times). A related study on Indigenous patients perspectives on follow-up cancer care also found that flexible care responsive to patients’ needs was essential in increasing service accessibility [30]. The use of innovative approaches such as telehealth that minimises patient travel was found to be an enabler to accessing cancer care in this study, as has been found in other studies [5,30,61]. For example, the use of a telehealth model to provide specialist medical oncology care to rural patients in North Queensland has allowed patients to receive cancer treatment closer to their homes, be reviewed by medical oncologists for complications in a timely manner and have better access to specialist care [62].

The recommended strategies provided by participants in this study (see Tables 2 and 3) also align with a number of WHO identified actionable priorities to enhance continuity and coordination of care [26]. These strategies include: collaborative planning and shared decision making (relating to follow-up cancer care in this study); case management for people with complex needs (care coordination identified as important in this study); comprehensive care along the entire pathway (the need to address patients’ needs and for flexibility in care delivery); and the use of technology to support continuity and coordination of care (participants in this study recommended sharing of EMRs across services) [26].

The WHO IPCHS framework was used in this paper to show the pathway for each IPCHS primary driver and how the various types of contributions and participant recommended strategies could lead to delivering a desired outcome. Together, these primary drivers should contribute to achieving seamless continuity and well-coordinated care within and across health settings and sectors which in turn should contribute towards integrated people- centred health services [25].

**Figure 1:**
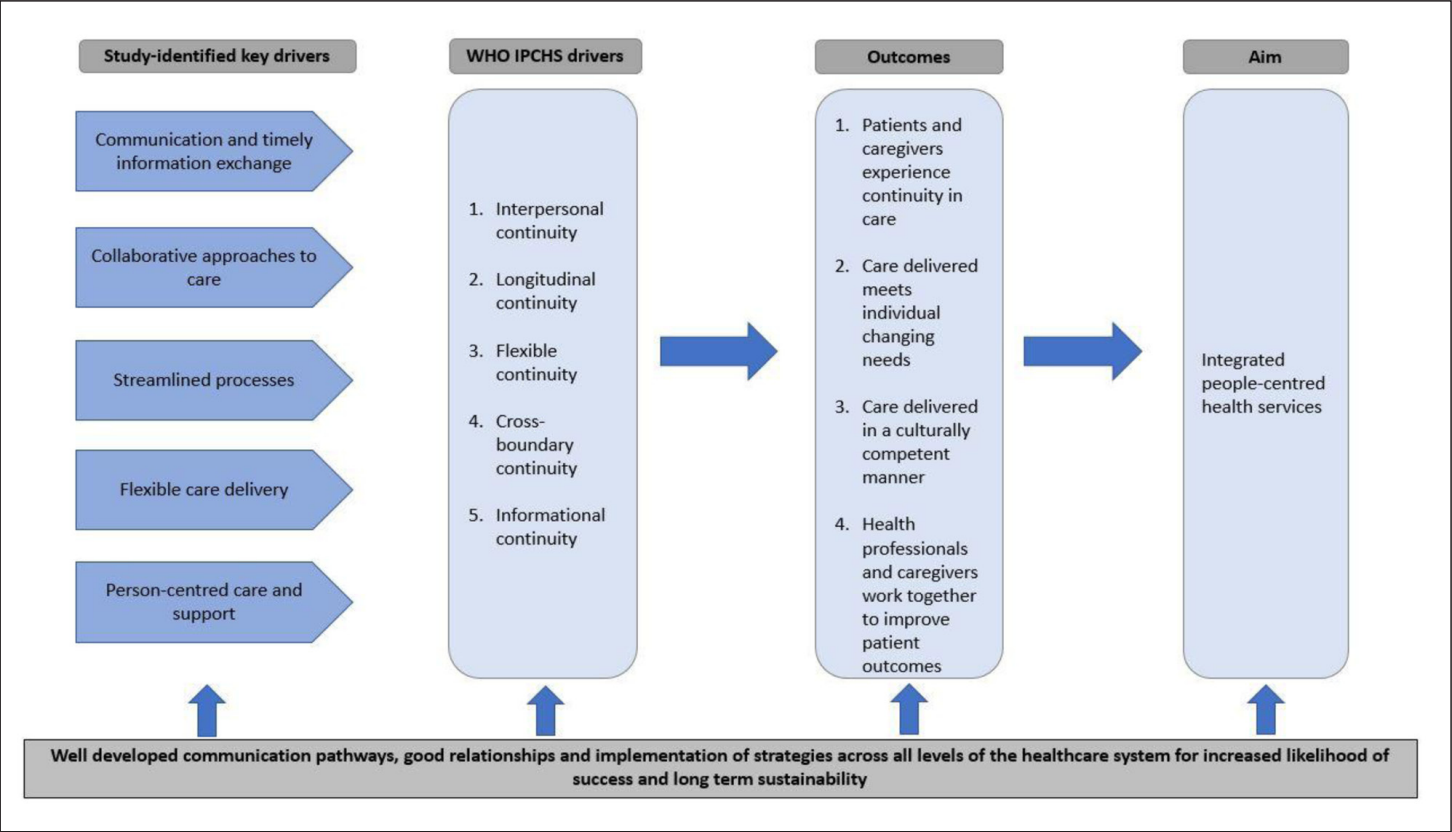
Drivers of continuity and care coordination from this study added to the implementation guide of the WHO Framework of Integrated People-Centred Health Services [26].

Insights from our study findings have enabled us to suggest several additions to the implementation of the IPCHS framework (Figure 1). Firstly, by adding an additional desired outcome, that care is delivered in a culturally competent manner. This outcome is to ensure that the framework is tailored to address the needs of Indigenous people diagnosed with cancer with the aim of increasing accessibility of services and improving outcomes. Secondly, the key drivers identified in this study (the major findings) have been added to the framework. As shown in Figure 1, these key drivers feed into the WHO drivers (i.e. the various types of continuity) to achieve the desired outcomes. Thirdly, underpinning all these steps and based on study findings, the authors have highlighted the need for well-developed communication pathways, good collaborative relationships between service providers, and the need for a whole-of-systems approach to increase the likelihood of success in implementing strategies and long-term sustainability. This multilevel approach requires support and commitment from all players including individuals, care providers and teams, service organisations and their leaders, within a supportive broader health policy environment [63]. It is anticipated that when desired outcomes are achieved, patients experience smooth transitions through the various stages of care across settings, care is well coordinated and effective, provided in a culturally competent manner, is provided based on patients changing needs and contributes to improved health system performance [26].

## Strengths and weaknesses

This study included a small number of Indigenous PHC services and one public-sector cancer treating hospital. Care needs to be exercised in generalising the study results to the wider range of services for Indigenous Australians with cancer. It is recommended that further research be conducted with larger samples across settings and geographical locations. However, this is an important study which was carried out with methodological rigour in the collection and analysis of data. It provides insight into experiences and strategies that several services utilise and reflects the recommendations of participants and the authors (based on study findings) to improve the continuity and coordination of care between services and settings to provide seamless quality care for Indigenous cancer patients in Queensland.

## Implications of the findings for policy, practice and further research

Our findings suggest that there is significant room for improvement in the delivery of continuous and coordinated cancer care for Indigenous people in Queensland. Effective communication strategies, timely information exchange, collaborative approaches to care, streamlined processes enabling the sharing of patient information, patient-centred care and support, and the flexible delivery of care between providers across settings were identified as areas for further development. Organisational and health system policies, protocols and guidelines that support people and systems to promote effective communication and information exchange processes are recommended for the ongoing provision of continuous and coordinated care between services and across settings. Enabling all registered and treating service providers across settings to have shared access to their patients’ electronic records could improve the provision of continuous and coordinated care for cancer patients. While the sharing of patient information records with relevant care providers may deliver quality and continuous care, it is important to ensure that patient confidentiality and privacy are respected and strictly upheld. Further research exploring Indigenous patients’ perspectives on sharing of their health-related information with multiple providers, and exploring patient consenting options across sites and services is highly recommended.

## Conclusions

Strong partnerships and collaboration between service providers across primary health care and hospital settings and good communication are integral to delivering continuous and coordinated cancer care for Indigenous Australians. Strategies to enhance communication and the sharing and timely exchange of patient information within and between services need to be strengthened, while ensuring patient confidentiality continues to be upheld and respected. Strong commitment from governments, supportive health policies, and appropriate models of health funding that encourage and reward collaborative and partnership approaches within and across settings that prioritise improved patient outcomes are crucial in the provision of seamless quality cancer care for Indigenous people in Queensland, Australia.
