# Communication, Collaboration and Care Coordination: The Three-Point Guide to Cancer Care Provision for Aboriginal and Torres Strait Islander Australians

**DOI:** 10.5334/ijic.5641

**Published:** 2020-12-01

**Authors:** Audra de Witt, Veronica Matthews, Ross Bailie, Gail Garvey, Patricia C. Valery, Jon Adams, Jennifer H. Martin, Frances C. Cunningham

**Affiliations:** 1 Menzies School of Health Research, Brisbane QLD, Charles Darwin University, Darwin NT, AU; 2 QIMR Berghofer Medical Research Institute, Brisbane QLD, AU; 3 University Centre for Rural Health, University of Sydney, NSW, AU; 4 Faculty of Health, University of Technology Sydney, Sydney NSW, AU; 5 School of Medicine and Public Health, University of Newcastle, Callaghan NSW, AU; 6 Southside Clinical School, University of Queensland, Brisbane QLD, AU

**Keywords:** indigenous people, cancer care coordination, integrated care, collaboration, communication, primary health care

## Abstract

This article details a correction to the article: de Witt A, Matthews V, Bailie R, Garvey G, Valery PC, Adams J, et al. Communication, Collaboration and Care Coordination: The Three-Point Guide to Cancer Care Provision for Aboriginal and Torres Strait Islander Australians. International Journal of Integrated Care. 2020; 20(2): 10. DOI: https://doi.org/10.5334/ijic.5456.

## Correction

de Witt et al. (2020) [1] was published with a typesetting error in the Results section. The original text states:

“Participant-Identified barriers (across sites) included limited knowledge of some staff on qualitative study, was use cultural and clinical aspects of health care delivery for Indigenous people.”

This sentence should instead read:

“Participant-Identified barriers (across sites) included limited knowledge of some staff on cultural and clinical aspects of health care delivery for Indigenous people.”

This error was introduced by the publisher during typesetting.
